# A proposed division of the family *Picornaviridae* into subfamilies based on phylogenetic relationships and functional genomic organization

**DOI:** 10.1007/s00705-021-05178-9

**Published:** 2021-08-04

**Authors:** Roland Zell, Nick J. Knowles, Peter Simmonds

**Affiliations:** 1 Section of Experimental Virology, Institute for Medical Microbiology, Jena University Hospital, Friedrich Schiller University, Hans-Knoell-Str. 2, 07745 Jena, Germany; 2 The Pirbright Institute, Ash Road, Pirbright, Woking, Surrey, GU24 ONF UK; 3 Nuffield Department of Medicine, University of Oxford, Peter Medawar Building, South Parks Road, Oxford, OX1 3SY UK

## Abstract

The highly diverse virus family *Picornaviridae* presently comprises 68 approved genera with 158 species plus many unassigned viruses. In order to better match picornavirus taxonomy to the functional and genomic groupings between genera, the establishment of five subfamilies (*Caphthovirinae, Kodimesavirinae, Ensavirinae, Paavivirinae* and *Heptrevirinae*) is proposed. The subfamilies are defined by phylogenetic analyses of 3CD (precursor of virus-encoded proteinase and polymerase) and P1 (capsid protein precursor) coding sequences and comprise between 7 and 22 currently approved virus genera. Due to the high within-subfamily and between-subfamily divergences of the picornavirus genera, p-distance estimates are unsuited for the demarcation of subfamilies. Members of the proposed subfamilies typically show some commonalities in their genome organisations, including VP1/2A cleavage mechanisms and possession of leader proteins. Other features, such as internal ribosomal entry site types, are more variable within and between members of genera. Some subfamilies are characterised by homology of proteins 1A, 2A, 2B and 3A encoded by members, which do not belong to the canon of orthologous picornavirus proteins. The proposed addition of a subfamily layer to the taxonomy of picornaviruses provides a valuable additional organisational level to the family that acknowledges the existence of higher-level evolutionary groupings of its component genera.

**Supplementary Information:**

The online version contains supplementary material available at 10.1007/s00705-021-05178-9.

## Introduction

Viruses of the family *Picornaviridae* have small (~30 nm), non-enveloped capsids with T=1/pseudo T=3 symmetry [11]. Their RNA genomes have positive-strand polarity and lengths that range from 6.7 to 10.1 kb [13]. With the exception of dicipiviruses, all picornavirus genomes possess a single open reading frame (ORF) that is flanked on both sides by untranslated regions (UTRs) with signal structures for the initiation of translation and replication. The ORF encodes a polyprotein that is co- and post-translationally processed by one to three virus-encoded proteinases. Translation is directed by one of five known types of internal ribosome entry site (IRES). Structural proteins (capsid proteins, CPs) are located in the N-terminal part of the polyprotein. The nonstructural proteins function in virus replication and include 2C^hel^, a helicase with a typical fold of P-loop ATPases, 3B^VPg^, a small genome-linked oligopeptide, 3C^pro^, a chymotrypsin-like cysteine proteinase, and 3D^pol^, an RNA-dependent RNA polymerase (RdRP) (for a recent review, see ref. [12]). Dicipiviruses are the only known picornaviruses with a dicistronic genome. ORF1 encodes the CP precursor, whereas ORF2 yields the non-structural proteins. Here, translation of both polyproteins is facilitated by two separate IRESs. The CP domains and the Hel-Pro-Pol domains of the polyprotein are known as the 'CP module' and the 'Hel-Pro-Pol core replicative module' or as the 'Hel-Pro-Pol replication block' and are characteristic of all members of the order *Picornavirales* [3, 9].

Picornaviruses are genetically highly diverse and infect members of six vertebrate classes on all continents. Members of the family *Picornaviridae* are currently classified into 68 assigned genera with 158 species. However, many yet unassigned viruses are awaiting classification. All picornaviruses share orthologous proteins, which exhibit the characteristic picornavirus hallmarks (i.e., rhv domains with a jelly roll fold of the three major CPs, a Walker A motif of 2C^hel^, a GxCGx_10-15_GxH active site sequence of the 3C^pro^, and the RdRP sequence motifs KDE, DxxxxD, (Y)GDD and FLK(R) of the 3D^pol^). More-variable and often genetically non-homologous picornavirus proteins include the leader protein (L), where present, 1A, 2A, 2B, 3A and 3B, although some may play similar functional roles in replication.

Picornavirus genera are distinguished on the basis of genetic relationships. Members of a given genus share significant sequence similarity (usually greater than 40% amino acid sequence identity in 3D^pol^) and cluster together in phylogenetic analysis. In general, they also share a common – although not invariant – genome layout. An idealised genome layout, the L434 schema, was proposed by Rueckert and Wimmer [8]. This describes a genome encoding a polyprotein that is post- or co-translationally cleaved into a leader (non-structural) protein, a structural gene block (P1) that is further cleaved into four protein components of the nucleocapsid, and two blocks of non-structural proteins (P2, P3) typically cleaved into three and four component functional proteins, respectively. However, several exceptions have been reported, including the variable presence of an L protein, variation in the numbers of 2A and 3B (VPg) proteins, differences in the mechanism of cleavage of VP1 from 2A, and a lack of cleavage of the VP0 protein (resulting in VP4 and VP2) in the mature nucleocapsid of some picornaviruses, such as parechoviruses.

As the number of classified genera in the family increased over time, five groups of picornavirus genera became evident in phylogenetic analyses. These groups of closely related genera were designed as “supergroups 1-5” (SG1-5) (N.J. Knowles at www.picornaviridae.com; [12]), but they never became part of the official picornavirus taxonomy. Novel highly divergent picornaviruses imply the existence of further, as yet undiscovered, ones.

Here, we investigate the potential taxonomic utility of the 'supergroup' concept and accordingly propose the establishment of five picornavirus subfamilies to be named *Caphthovirinae, Kodimesavirinae, Ensavirinae, Paavivirinae*, and *Heptrevirinae*. The results indicate that the picornavirus 'supergroups' based on analysis of the proteinase/polymerase precursor 3CD are useful to define the taxonomic rank of picornavirus subfamilies. These proposed assignments to subfamilies have the additional value of defining virus groups with distinct features of their genome layouts and replication strategies.

## Materials and methods

Genome sequences of viruses belonging to 158 currently recognised species of 68 picornavirus genera were retrieved from the GenBank database and aligned using MEGA X [4]. Alignments were adjusted manually. For phylogenetic analysis, gene regions corresponding to P1 (capsid proteins), 2A, 2B, 3A, and 3CD (precursor of virus-encoded proteinase and polymerase) were used to infer phylogenetic trees with MrBayes v.3.2 [7]. The best-fit substitution model was selected using the Find Best DNA/Protein Models option implemented in MEGA. Phylogenetic trees were visualized with FigTree v1.4.4 (http://tree.bio.ed.ac.uk/software/figtree). Divergence was estimated using MEGA X. Results obtained using GRAViTy (http://gravity.cvr.gla.ac.uk; [1]) were compared with those obtained by phylogenetic analysis of 3CD and P1.

## Results

### Phylogeny of 3CD-encoding sequences identifies clusters that delineate the proposed subfamilies

**Fig. 1:**
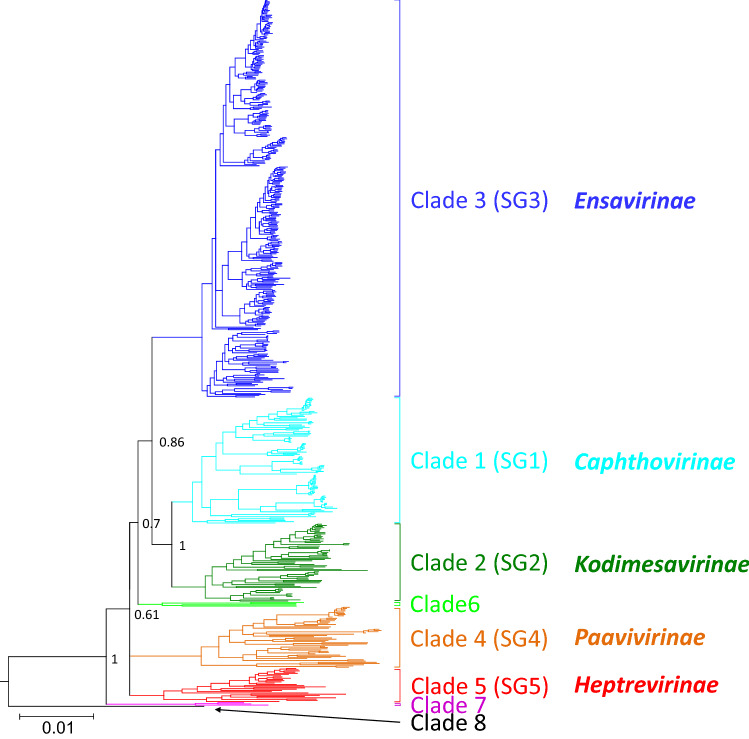
Phylogenetic analysis based on the picornavirus 3CD protein. A total of 578 sequences representing the 3CD region (3162 nt) of members of all known picornavirus species and types were analysed with MrBayes v3.2 (nucleotide substitution model HKY+G+I). Convergence was reached after 13 million generations. Sequences cluster in clades 1 to 8 (indicated in different colors). Clades 1 to 5 correspond to supergroups (SGs). Posterior probabilities of major clades are presented. The scale indicates substitutions per nucleotide.

Phylogenetic analysis of the 3CD-encoding region of 578 sequences, each representing a distinct (geno)type of 158 currently accepted picornavirus species revealed eight clades (comprising between one and 354 sequences). Five clades correspond to the previously defined picornavirus “supergroups”, while further lineages (clades 6-8) incorporate the recently described (i) harkavirus A1 and three yet unassigned picornaviruses (GenBank accession numbers MG600082, MG600084, and MG600085), (ii) three currently unclassified reptile- and primate-derived picornaviruses (GenBank accession numbers MF370941, MG600088, and MG600104), and (iii) ampivirus A1 [2, 6, 10]. All clades are supported by posterior probabilities greater than 0.6 (Fig. 1; details are presented in Supplementary Fig. S1).

### P1-encoding sequences of members of the proposed subfamilies cluster in phylogenetic analysis

**Fig. 2:**
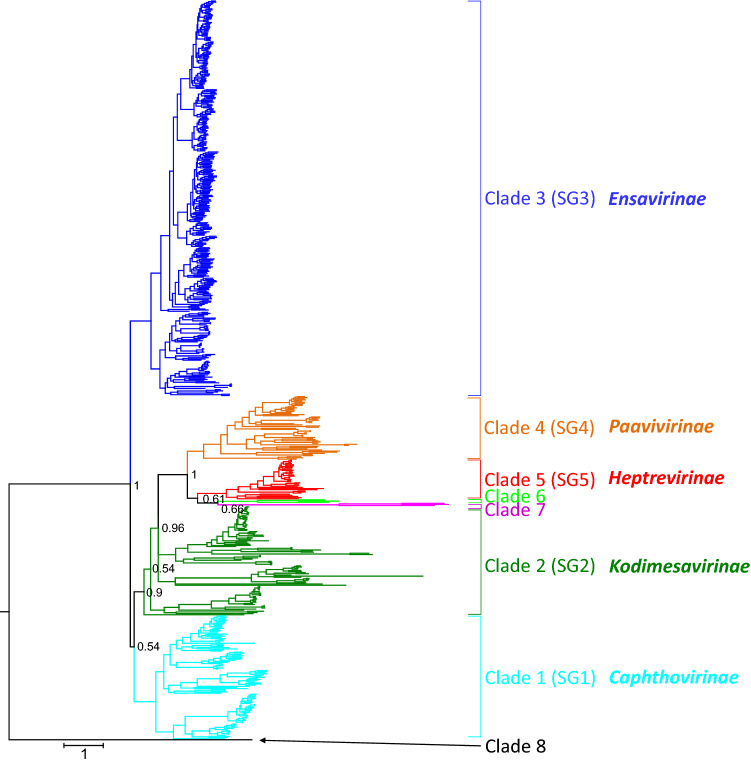
Phylogenetic analysis based on the picornavirus P1 protein. A total of 651 sequences representing the P1 region (5214 nt) of members of all known picornavirus species and types were analysed with MrBayes v3.2 (nucleotide substitution model GTR+G+I). Convergence was reached after 17 million generations. Sequences cluster in clades (indicated in different colors). Clades 1 to 5 correspond to supergroups. Posterior probabilities of major clades are presented. The scale indicates substitutions per nucleotide.

A phylogenetic analysis of the CP-encoding gene region (P1) yielded clades with significant support corresponding to SG1, SG3, SG4, and SG5. Only SG2 lacked monophyly, but all of the nodes showed low posterior probability, indicating poor robustness of the tree. Two additional clades identified in 3CD analysis containing the sequences of harkavirus A1 and the six unassigned picornaviruses showed a similarly distinct clustering in P1 (Fig. 2; details in Supplementary, Fig. S2). As in the 3CD-based tree, the ampivirus sequence branched separately.

### Genomic and functional properties of the supergroups and proposed picornavirus subfamilies

**Fig. 3:**
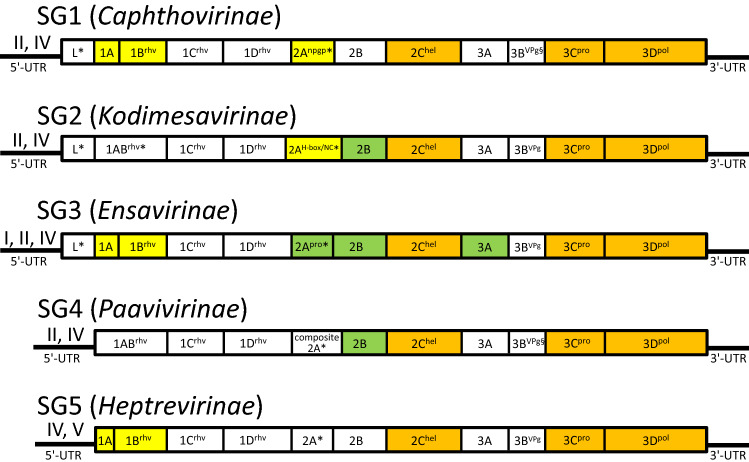
Schematic presentation of picornaviral genome organization and expression (not drawn to scale). Five supergroups (SGs) are distinguished by phylogenetic relationships (compare Figures 1 and 2) and different functional organisation of their genomes. The open reading frame encoding a polyprotein is indicated by a box. Only dicipiviruses of the proposed subfamily *Kodimesavirinae* have dicistronic genomes (not shown). Localisation of processed proteins in the polyprotein is indicated. Protein designations follow the L434 rule. Members of the *Paavivirinae* and the megriviruses of the *Kodimesavirinae* may have a composite 2A gene region comprising up to six 2A domains with partly unknown functions. Superscripts indicate domains with known function, i.e., rhv, characteristic jelly roll folding of capsid proteins, pro, chymotrypsin-like cysteine proteinase domain; npgp, cis-active translational termination-reinitiation site; H-box/NC, a domain with similarity to the H-rev 107 family of proteins; hel, P-loop ATPase-like helicase domain; VPg, genome-linked viral peptide; pol, RNA-dependent RNA polymerase. * indicates genome regions that are not shared by all members of the respective supergroup/subfamily (the predominant domain is shown). § indicates that the copy number of 3B^VPg^ peptide may vary in a few members. Roman numerals indicate IRES types found in the 5' untranslated region (5'-UTR). Orange boxes indicate the proteins of the Hel-Pro-Pol core replicative module that are common to all picornaviruses, green boxes indicate proteins that are orthologous within the respective supergroup/subfamily only, and yellow boxes highlight specific features characteristic of some supergroups/subfamilies.

***Caphthovirinae* (SG1)**. The name was derived from the two earliest described genera ***Ca****rdiovirus* and *A****phtho****virus*. This subfamily includes the genera *Ailurivirus, Aphthovirus, Bopivirus, Cardiovirus, Cosavirus, Erbovirus, Hunnivirus, Malagasivirus, Mischivirus, Mosavirus, Mupivirus, Senecavirus, Teschovirus, Torchivirus, Tottorivirus*, and *Marsupivirus* (35 species). A characteristic feature of virus genome expression in this subfamily is the cleavage of the CP precursor 1AB (VP0) into 1A (VP4) and 1B (VP2) (Fig. 3). Other typical but not invariant features are the presence of a leader protein (exceptions: *Bopivirus, Cosavirus, Marsupivirus*) and a 2A protein with an NPG↓P motif (exceptions: *Cardiovirus C, Malagasivirus, Mupivirus, Tottorivirus*). Aphthoviruses and erboviruses possess a leader proteinase (L^pro^). Mosaviruses have two copies of 3B^VPg^, while foot-and-mouth disease virus (genus *Aphthovirus*) has three copies. Viruses in the majority of genera possess a type II IRES.

***Kodimesavirinae* (SG2)**. The name was derived from the four earliest described genera of this subfamily, ***Ko****buvirus*, ***Di****cipivirus*, ***Me****grivirus*, and ***Sa****livirus*. This subfamily includes the genera *Danipivirus, Dicipivirus, Gallivirus, Hemipivirus, Kobuvirus, Livupivirus, Ludopivirus, Megrivirus, Myrropivirus, Oscivirus, Passerivirus, Pemapivirus, Poecivirus, Pygoscepivirus, Rafivirus, Rajidapivirus, Rosavirus, Sakobuvirus, Salivirus, Sicinivirus, Symapivirus*, and *Tropivirus* (38 species). A characteristic feature is that the 2B protein is conserved (orthologous) among the members of the subfamily (Fig. 3, Supplementary Fig. S3). Other typical features of most members of this subfamily are a leader protein (exceptions: *Dicipivirus, Megrivirus A*, *Megrivirus B*, *Megrivirus C*, *Megrivirus D, Poecivirus, Rafivirus C, Rosavirus*), an uncleaved 1AB (exceptions: *Danipivirus, Dicipivirus, Rosavirus*), the presence of a 2A protein with an H-box/NC motif (exceptions: *Dicipivirus*, a few kobuviruses, *Oscivirus, Pygoscepivirus* and *Rajidapivirus*; some megriviruses exhibit a composite 2A gene region with three different 2A proteins, the last of which has a H-box/NC motif). Viruses of the *Kodimesavirinae* have a type II or type IV IRES.

***Ensavirinae* (SG3)**. The name was derived from the two earliest described genera, ***En****terovirus* and ***Sa****pelovirus*. This subfamily includes the genera *Anativirus, Boosepivirus, Diresapivirus, Enterovirus, Felipivirus, Parabovirus, Rabovirus,* and *Sapelovirus* (30 species). Characteristic features are the cleavage of 1AB and the presence of a 2A protein with proven or assumed chymotrypsin-like cysteine proteinase activity (Fig. 3; exception: *Anativirus*, whose members appear to lack a functional 2A protein). Besides the 1A and 2A proteins, the 2B and 3A proteins are also conserved (orthologous) among the members of this subfamily (Supplementary Figs. S4, S5, and S6). IRES types I, II and IV are found.

***Paavivirinae* (SG4)**. The name was derived from the two earliest described genera, ***Pa****rechovirus* and ***Avi****hepatovirus*. This subfamily includes the genera *Aalivirus, Aquamavirus, Avihepatovirus, Avisivirus, Crohivirus, Grusopivirus, Kunsagivirus, Limnipivirus, Orivirus, Parechovirus, Pasivirus, Potamipivirus,* and *Shanbavirus* (29 species). A characteristic feature of all members is that the polyprotein lacks an L protein and that 1AB remains uncleaved (Fig. 3). The 2B protein is conserved (orthologous) among all members of this subfamily (Supplementary Fig. S7). Viruses of this subfamily exhibit the highest variability in their 2A gene region. Most viruses have one of three composite 2A gene regions: (i) one to three 2A proteins with an NPG↓P motif plus a protein with an H-box/NC motif (crohiviruses, grusopi A viruses, parechoviruses B-F, potamipiviruses, and shanbaviruses), (ii) one to four 2A proteins with an NPG↓P motif plus a domain with homology to the AIG1-type guanine nucleotide-binding domain plus a domain with an H-box/NC motif (aaliviruses, avihepatoviruses and avisiviruses), (iii) one to three 2A proteins with an NPG↓P motif plus a unique protein domain with unknown function (aquamaviruses and kunsagiviruses). A few viruses of the subfamily have single 2A proteins: (i) members of the species *Parechovirus A* have only a protein with H-box/NC motif, (ii) pasiviruses have a protein with an NPG↓P motif, and (iii) oriviruses and grusopi B viruses have a 2A protein with unknown function. All known aquamaviruses have two copies of 3B^VPg^. The IRES belongs to type II or IV.

***Heptrevirinae* (SG5)**. The name was derived from the two earliest described genera, ***Hep****atovirus* and ***Tre****movirus*. This subfamily includes the genera *Caecilivirus, Crahelivirus, Fipivirus, Gruhelivirus, Hepatovirus, Rohelivirus,* and *Tremovirus* (21 species). Characteristic features include a very short 1A peptide (14-35 amino acids) and the occurrence of 1AB cleavage (Fig. 3). The 2A protein in hepatoviruses has an unknown function and is believed to be cleaved from the VP1-2A precursor (pX) by an unknown host proteinase at a late stage of virion morphogenesis. Other viruses of the subfamily (caeciliviruses, craheliviruses, gruheliviruses, and roheliviruses) may apply a similar mechanism. Fipiviruses and tremoviruses have 2A proteins with an H-box/NC motif. Hepatoviruses have a type V IRES, and tremoviruses have a type IV IRES. The IRES type of the remaining viruses of this subfamily is undetermined.

**Picornaviruses not assigned to a subfamily.** In addition to the many picornavirus sequences assigned to the five proposed subfamilies, highly divergent virus sequences of the genera *Ampivirus* and *Harkavirus* as well as several further currently unclassified picornaviruses are candidates for inclusion in additional subfamilies. However, the limited availability of sequence data for them prevents reliable assignment to one of the proposed subfamilies or the definition of a new subfamily.

### Divergence estimates

‘Supergroups' 1 to 5 comprised between 7 and 22 picornavirus genera, whereas clade 6 consisted of harkavirus A1 and three unassigned viruses, SG7 of three yet unassigned viruses, and clade 8 only of ampivirus A1. Long branch lengths of the unassigned members of clades 6 and 7 in the phylogenetic trees (Figs. 1, and 2, Supplementary Figs. S1 and S2) suggest that they belong to novel genera.

The mean *within-group* divergence of the 3CD amino acid sequences of the five 'supergroups' ranges from 0.408 (S.E. 0.012) to 0.639 (S.E. 0.01), whereas the *between-group* divergence is greater 0.7 (S.E. 0.01-0.013) (Table 1). The mean *within-group* divergence values of the P1 amino acid sequences (Table 2) are less than 0.714 (S.E. 0.06-0.007), whereas the mean *between-group* divergence values exceed 0.76 (S.E. 0.007-0.009).

**Table 1:** Estimates of evolutionary divergence over 3CD sequence pairs within and between groups (mean p-distances ± S.E.)

	SG1	SG2	SG3	SG4	SG5
SG1	**0.590 ± 0.010**				
SG2	0.724 **± **0.011	**0.575 ± 0.011**			
SG3	0.727 **± **0.012	0.707 **± **0.013	**0.408 ± 0.012**		
SG4	0.801 **± **0.010	0.793 **± **0.011	0.772 **± **0.012	**0.639 ± 0.010**	
SG5	0.791 **± **0.010	0.786 **± **0.011	0.767 **± **0.011	0.808 **± **0.010	**0.624 ± 0.009**

p-distance, number of amino acid substitutions per site; S.E., standard error; SG1, *Caphthovirinae*; SG2, *Kodimesavirinae*; SG3, *Ensavirinae*; SG4, *Paavivirinae*; SG5, *Heptrevirinae*

**Table 2:** Estimates of evolutionary divergence over P1 sequence pairs within and between groups (mean p-distances **±** S.E.)

	SG1	SG2	SG3	SG4	SG5
SG1	**0.649±0.009**				
SG2	0.800**±**0.007	**0.714±0.0 06**			
SG3	0.765**±**0.009	0.801**±**0.008	**0.530±0.009**		
SG4	0.847**±**0.008	0.854**±**0.007	0.858**±**0.008	**0.688±0.007**	
SG5	0.851**±**0.009	0.854**±**0.008	0.852**±**0.009	0.838**±**0.008	**0.522±0.008**

p-distance, number of amino acid substitutions per site; S.E., standard error; SG1, *Caphthovirinae*; SG2, *Kodimesavirinae*; SG3, *Ensavirinae*; SG4, *Paavivirinae*; SG5, *Heptrevirinae*

Comparisons of averaged *within-group* and *between-group* divergence yield results that allow the distinction of five 'supergroups' corresponding to the respective clades shown in Figure 1. The frequency distribution of pairwise amino acid sequence identity scores, however, indicates substantial overlaps for P1, 3CD and 3D comparisons (Supplementary Fig. S8). The results suggest that p-distance estimates are an unsuitable tool for describing possible picornavirus subfamilies.

### Analysis of whole-genome relationships using GRAViTy

Sequences representative of each classified picornavirus from the ICTV Virus Metadata Resource (https://talk.ictvonline.org/taxonomy/vmr/) along with a compilation of 75 unassigned full-length candidate picornavirus sequences representing members of eight clades shown in Fig. 1 were analysed using the GRAViTy server (http://gravity.cvr.gla.ac.uk). The program (“Genome Relationships Applied to Virus Taxonomy") compared the picornavirus sequences with all full genome sequences of currently classified +stranded viruses through computation of composite generalized Jaccard (CGJ) distances derived from hidden Markov model profile similarities of translated sequences, a metric derived from similarities in genome organizational features (gene orders and orientations) [1].

**Fig. 4:**
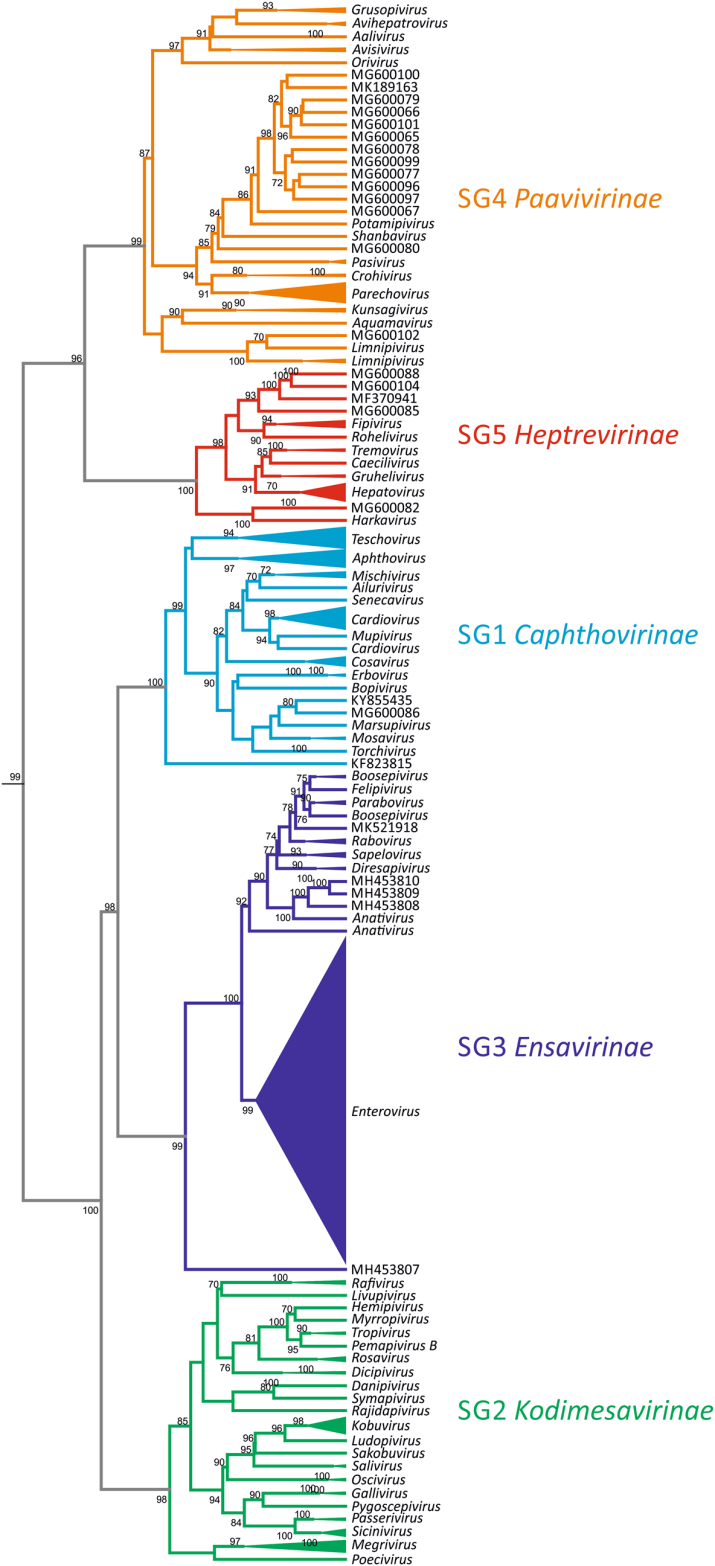
GRAViTy analysis. Output from analysis by GRAViTy of 75 full-length candidate picornavirus sequences downloaded from GenBank and the RNA virus dataset (DB-4 - Baltimore Group III, IV, V, VI and VII - all RNA viruses and retroelements), using the web server hosted by the MRC-University of Glasgow (http://gravity.cvr.gla.ac.un). The section of the dendrogram containing the submitted sequences and classified picornaviruses within DB-4 is shown (with the exception of ampivirus, which grouped elsewhere in the *Picornavirales* clade). Branches representing existing and proposed genera are condensed. Unassigned viruses are indicated by GenBank accession numbers. Booster bootstrap values [5] of ≥70% generated from 100 data resamplings are shown on branches. Picornavirus supergroups (SGs) are presented in different colours.

Picornaviruses formed a monophyletic group in a dendrogram constructed from CGJ distances (Fig. 4), with the exception of ampiviruses (clade 8), which grouped in a clade containing seco-, ifla-, polycipi-, solinvi-, marna- and dicistroviruses (data not shown). Within the picornavirus-specific clade, the five main groupings corresponded to the proposed subfamilies *Caphthovirinae, Kodimesavirinae, Ensavirinae, Paavivirinae* and *Heptrevirinae* (Fig. 4). Each subfamily-associated cluster was defined by a relatively long, bootstrap-supported branch in the dendrogram. However, picornavirus sequences assigned to clades 6 and 7 clustered within the subfamily *Heptrevirinae* (SG5) and did not display the same differentiation from each other and other *Heptrevirinae* members as observed in other subfamilies. Furthermore, Fujian spotted paddle-tail newt picornavirus (MG600085), a proposed member of clade 6, failed to cluster with the two other picornaviruses assigned to this clade. Apart from these two exceptions, there was a close correlation in branching order and branching lengths between genome relationships inferred by GRAViTy with those obtained by phylogenetic analysis of P1 and 3CD regions for all five proposed subfamilies, and almost all genera and species.

## Discussion

The available sequence data indicate well-distinguished groups of related picornavirus genera as defined by the monophyly of 3CD sequence clusters in phylogenetic analyses (Fig. 1). With the exception of ampiviruses, with only one available sequence, and the proposed subfamily *Kodimesavirinae*, the P1 sequences of the members of all remaining subfamilies also cluster in clades comprising at least three sequences (Fig. 2). These clades correspond to five previously defined picornavirus 'supergroups' and allow the identification of three possible additional supergroups. However, while the members of the subfamily *Kodimesavirinae* formed a monophyletic cluster in the phylogenetic analysis of the 3CD region (Fig. 1) and in analysis by GRAViTy (Fig. 4), the proposed subfamily was polyphyletic in the P1 region (Fig. 2). At present, it is unclear whether this is the result of a degree of mosaicism through recombination affecting the structural gene region or of an artefact from alignment errors with highly divergent sequences. This question may be resolved with the availability of additional sequences from this subfamily in the future. It is potentially relevant that members of the *Kodimesavirinae* also show inconsistent clustering in phylogenetic analyses, which has partly been attributed to interspecies recombination events [12].

The estimation of p-distances, which proved to be helpful for the assignment of picornaviruses at the levels of types, species and genera, is of limited use for classification in the proposed subfamilies. Pairwise amino acid frequency distributions of P1, 3CD, and 3D proteins showed considerable overlaps in both *within-subfamily* and *between-subfamily* distance distributions (Supplementary Fig. S8). Hence, other criteria must be applied. The presence or absence of one or more non-structural proteins that are unique to individual genera could be an expedient alternative, such as the extremely short 1A protein of the members of the *Heptrevirinae*, the 2A^pro^ protein of the *Ensavirinae*, the 2B proteins of the members of the *Ensavirinae*, *Kodimesavirinae* and *Paavivirinae*, and the 3A of the *Ensavirinae*. Shared possession of such proteins within a subfamily provides strong support for their evolutionary relatedness to each other. Indeed, phylogenetic analysis of the 2A^pro^ of the *Ensavirinae*, the 2B protein of the *Kodimesavirinae*, *Ensavirinae*, and *Paavivirinae*, and the 3A protein of the *Ensavirinae* (Supplementary Figs. S3-S7) yields tree topologies that resemble those of the respective 3CD trees (data not shown). The recognition that several picornavirus proteins in different subfamilies may have separate origins and share no sequence homology is relevant for the selection of genome regions for virus classification – degrees of similarity computed between sequences in alignments that are non-homologous are actually meaningless and should be excluded from phylogenetic comparisons.

GRAViTy, an analysis pipeline that has been developed as an alignment-free tool to evaluate virus groupings into families and orders, independently confirmed the existence of systematic genetic similarities and differences that defined the proposed picornavirus subfamilies. The dendrogram created from CGJ distances produced five clades and correctly assigned unclassified sequences to the corresponding subfamilies (compare Figures 1, 2, and 4). Interestingly, monophyly was clearly apparent for the viruses assigned to the subfamily *Kodimesavirinae* even though the P1 tree for these viruses was polyphyletic (compare Figures 2 and 4). The GRAViTy results based on whole-genome features strongly supports the proposal advanced in the current study that picornavirus subfamilies indeed represent evolutionarily distinct groups of viruses, a conclusion supported by their possession of specific gene complements and other subfamily-specific genome organizational features.

The main anomaly between analysis methods was the evidence from analysis of the 3CD region for the existence of further supergroups of picornavirus genera, clades 6, 7, and 8 (Fig. 1), whereas their component sequences grouped clades 6 and 7 within SG5 (*Heptrevirinae*) in the P1 phylogeny and in GRAViTy analysis (Fig. 2 and 4). This discrepancy may be the result of the limited availability of full genome sequences of clade 6 and 7 variants for bioinformatic analysis. In addition, other unique genome features, such as a second open reading frame in the genome of Fujian spotted paddle-tail newt picornavirus (MG600085), which encodes a 264-aa protein of unknown function, would have been picked up as a discrepant genome organizational feature by GRAViTy, leading it to be separated from the two other sequences assigned to clade 6. Mosaicism may similarly account for the conflicting genomic relationships of the ampivirus sequences to those of picornaviruses. While its 3CD-encoding sequence places it at the root of the *Picornaviridae* clade, the low similarity of the third CP domain to the rhv domains of picornaviruses (pfam08762) accounts for its highly divergent placement in the P1 phylogenetic tree (Fig. 2) and its closer relationship to other *Picornavirales* members in the GRAViTy analysis. Unfortunately, the hierarchical taxonomy of viruses cannot accommodate mosaicism, and for viruses such as ampivirus, its final classification has to acknowledge this wider procedural limitation.

The formal establishment of picornavirus subfamilies that may replace the previously unofficially introduced 'supergroups' will increase the informativeness of picornavirus taxonomy. The family *Picornaviridae* is a fast-growing virus family, which currently includes 68 genera and 158 species with more than 670 distinct (geno)types. Future investigations may contribute to elaborating a fully consistent description of the subfamilies. At present, picornavirus subfamilies are defined by phylogenetic analysis of the 3CD protein and supported by additional phylogenetic analysis of the P1 polyprotein as well as whole genome analysis by GRAViTy and coherent features of CP, 2A, 2B, and 3A proteins.

An official taxonomic proposal for reorganization of the family *Picornaviridae* to include five new subfamilies described in this paper has been submitted on behalf of the Study Group to the ICTV for discussion and possible formal approval.

## Supplementary Information

Below is the link to the electronic supplementary material.

Supplementary file1Supplementary Fig. S1 Phylogenetic analysis based on picornavirus 3CD proteins. A total of 578 sequences representing the 3CD region (3162 nt) of members of all known picornavirus species and types were analysed with MrBayes v3.2 (nucleotide substitution model HKY+G+I). Convergence was reached after 13 million generations. Sequences cluster in clades 1 to 8 (indicated in different colors). Presented are GenBank accession number, species name (in bold and italics), virus name/type, common name (if available, in round brackets), and strain designation (in square brackets). Posterior probabilities of major clades are presented. The scale indicates substitutions per nucleotide (PDF 816 KB)

Supplementary file2Supplementary Fig. S2 Phylogenetic analysis of picornavirus P1 proteins. A total of 651 sequences representing the P1 region encoding the capsid proteins (5215 nt) of members of all known picornavirus species and types were analysed with MrBayes v3.2 (nucleotide substitution model GTR+G+I). Convergence was reached after 17 million generations. Sequences of clades 1 and 3 to 8 cluster in monophyletic clades (indicated in different colors). Presented are GenBank accession number, species name (in bold and italics), virus name/type, common name (if available, in round brackets), and strain designation (in square brackets). Posterior probabilities of major clades are presented. The scale indicates substitutions per nucleotide (PDF 787 KB)

Supplementary file3Supplementary Fig. S3 Phylogenetic analysis of supergroup 2 (*Kodimesavirinae*) 2B sequences. The alignment comprises 120 sequences of 22 genera, 38 species, 70 types, and 18 unassigned viruses. Tree inference was conducted with MrBayes, using the nucleotide substitution model GTR+G+I. Convergence was reached after 6 million generations. Unassigned viruses are shown in red. Presented are GenBank accession number, species name (in bold and italics), virus name/type, common name (if available, in round brackets), and strain designation (in square brackets) at the tips as well as posterior probabilities at the nodes. The scale indicates substitutions per nucleotide (PDF 295 KB)

Supplementary file4Supplementary Fig. S4 Phylogenetic analysis of supergroup 3 (*Ensavirinae*) 2A sequences. The alignment comprises 82 sequences of 8 genera, 30 species. Tree inference was conducted with MrBayes, using the nucleotide substitution model GTR+G+I. Convergence was reached after 7 million generations. Unassigned viruses are shown in red. Presented are GenBank accession number, species name (in bold and italics), virus name/type, common name (if available, in round brackets) and strain designation (in square brackets), at the tips as well as posterior probabilities at the nodes. The scale indicates substitutions per nucleotide (PDF 263 KB)

Supplementary file5Supplementary Fig. S5 Phylogenetic analysis of supergroup 3 (*Ensavirinae*) 2B sequences. The alignment comprises 89 sequences of 8 genera, 30 species. Tree inference was conducted with MrBayes, using the nucleotide substitution model GTR+G+I. Convergence was reached after 12 million generations. Unassigned viruses are shown in red. Presented are GenBank accession number, species name (in bold and italics), virus name/type, common name (if available, in round brackets) and strain designation (in square brackets), at the tips as well as posterior probabilities at the nodes. The scale indicates substitutions per nucleotide. Blue and green arrows indicate inconsistent clustering of boosepiviruses and paraboviruses, respectively (PDF 259 KB)

Supplementary file6Supplementary Fig. S6 Phylogenetic analysis of supergroup 3 (*Ensavirinae*) 3A sequences. The alignment comprises 90 sequences of 8 genera, 30 species. Tree inference was conducted with MrBayes, using the nucleotide substitution model GTR+G+I. Convergence was reached after 7 million generations. Unassigned viruses are shown in red. Presented are GenBank accession number, species name (in bold and italics), virus name/type, common name (if available, in round brackets), and strain designation (in square brackets) at the tips as well as posterior probabilities at the nodes. The scale indicates substitutions per nucleotide. Blue arrows indicate inconsistent clustering of parabo B and C viruses (PDF 266 KB)

Supplementary file7Supplementary Fig. S7 Phylogenetic analysis of supergroup 4 (*Paavivirinae*) 2B sequences. The alignment comprises 61 sequences of 13 accepted and proposed genera, 29 species. Tree inference was conducted with MrBayes, using the nucleotide substitution model GTR+G+I. Convergence was reached after 4 million generations. Unassigned viruses are shown in red. Presented are GenBank accession number, species name (in bold and italics), virus name/type, common name (if available, in round brackets), and strain designation (in square brackets) at the tips as well as posterior probabilities at the nodes. The scale indicates substitutions per nucleotide (PDF 245 KB)

Supplementary file8Supplementary Fig. S8 Frequency distribution of pairwise amino identity scores. P1, 3CD, and 3D proteins were compared. A total of 155 picornavirus P1 sequences and 156 3CD and 3D sequences were grouped into 68 genera. The data sets were completed with six sequences of unassigned viruses and were used for the estimation of pairwise amino acid identity scores (PDF 37 KB)

## Data Availability

Electronic supplementary material is included.

## References

[1] Aiewsakun P, Simmonds P (2018). The genomic underpinnings of eukaryotic virus taxonomy: creating a sequence-based framework for family-level virus classification. Microbiome.

[2] Boros A, Pankovics P, Simmonds P, Pollák E, Mátics R, Phan TG, Delwart E, Reuter G (2015). Genome analysis of a novel, highly divergent picornavirus from common kestrel (*Falco tinnunculus*): the first non-enteroviral picornavirus with type-I-like IRES. Inf Genet Evol.

[3] Le Gall O, Christian P, Fauquet CM, King AMQ, Knowles NJ, Nakashima N, Stanway G, Gorbalenya AE (2008). *Picornavirales*, a proposed order of positive-sense single-stranded RNA viruses with a pseudo-T = 3 virion architecture. Arch Virol.

[4] Kumar S, Stecher G, Li M, Knyaz C, Tamura K (2018). MEGA X: molecular evolutionary genetics analysis across computing platforms. Mol Biol Evol.

[5] Lemoine F, Domelevo Entfellner JB, Wilkinson E, Correira D, Davila Felipe M, De Oliveira T, Gascuel O (2018). Renewing Felsenstein’s phylogenetic bootstrap in the era of big data. Nature.

[6] Reuter G, Boros A, Tóth Z, Phan TG, Delwart E, Pankovics P (2015). A highly divergent picornavirus in an amphibian, the smooth newt (*Lissotriton vulgaris*). J Gen Virol.

[7] Ronquist F, Huelsenbeck JP (2003). MrBayes 3: Bayesian phylogenetic inference under mixed models. Bioinformatics.

[8] Rueckert R, Wimmer E (1984). Systematic nomenclature of picornavirus proteins. J Virol.

[9] Sanfaçon H, Gorbalenya AE, Knowles NJ, Chen YP, King AMQ, Adams MJ, Carstens EB, Lefkowitz EJ (2012). Order *Picornavirales*. Virus taxonomy. Ninth report of the international committee on taxonomy of viruses.

[10] Shi M, Lin XD, Chen X, Tian JH, Chen LJ, Li K, Wang W, Eden JS, Shen JJ, Liu L, Holmes EC, Zhang YZ (2018). the evolutionary history of vertebrate RNA viruses. Nature.

[11] Tuthill TJ, Groppelli E, Hogle JM, Rowlands DJ (2010). Picornaviruses. Curr Top Microbiol Immunol.

[12] Zell R (2018). *Picornaviridae*—the ever-growing virus family. Arch Viral.

[13] Zell R, Delwart E, Gorbalenya AE, Hovi T, King AMQ, Knowles NJ, Lindberg AM, Pallansch MA, Palmenberg AC, Reuter G, Simmonds P, Skern T, Stanway G, Yamashita T, ICTV Report Consortium (2017). ICTV virus Taxonomy Profile: *Picornaviridae*. J Gen Virol.

