# Supplementary material for: A proposed division of the family Picornaviridae into subfamilies based on phylogenetic relationships and functional genomic organization
Source: Arch Virol. 2021 Aug 4;166(10):2927–35. doi: 10.1007/s00705-021-05178-9 (PMC8421316; doi:10.1007/s00705-021-05178-9)
Supplement: Supplementary file 1 — Supplementary file1Supplementary Fig. S1 Phylogenetic analysis based on picornavirus 3CD proteins. A total of 578 sequences representing the 3CD region (3162 nt) of members of all known picornavirus species and types were analysed with MrBayes v3.2 (nucleotide substitution model HKY+G+I). Convergence was reached after 13 million generations. Sequences cluster in clades 1 to 8 (indicated in different colors). Presented are GenBank accession number, species name (in bold and italics), virus name/type, common name (if available, in round brackets), and strain designation (in square brackets). Posterior probabilities of major clades are presented. The scale indicates substitutions per nucleotide (PDF 816 KB) [file 705_2021_5178_MOESM1_ESM.pdf]

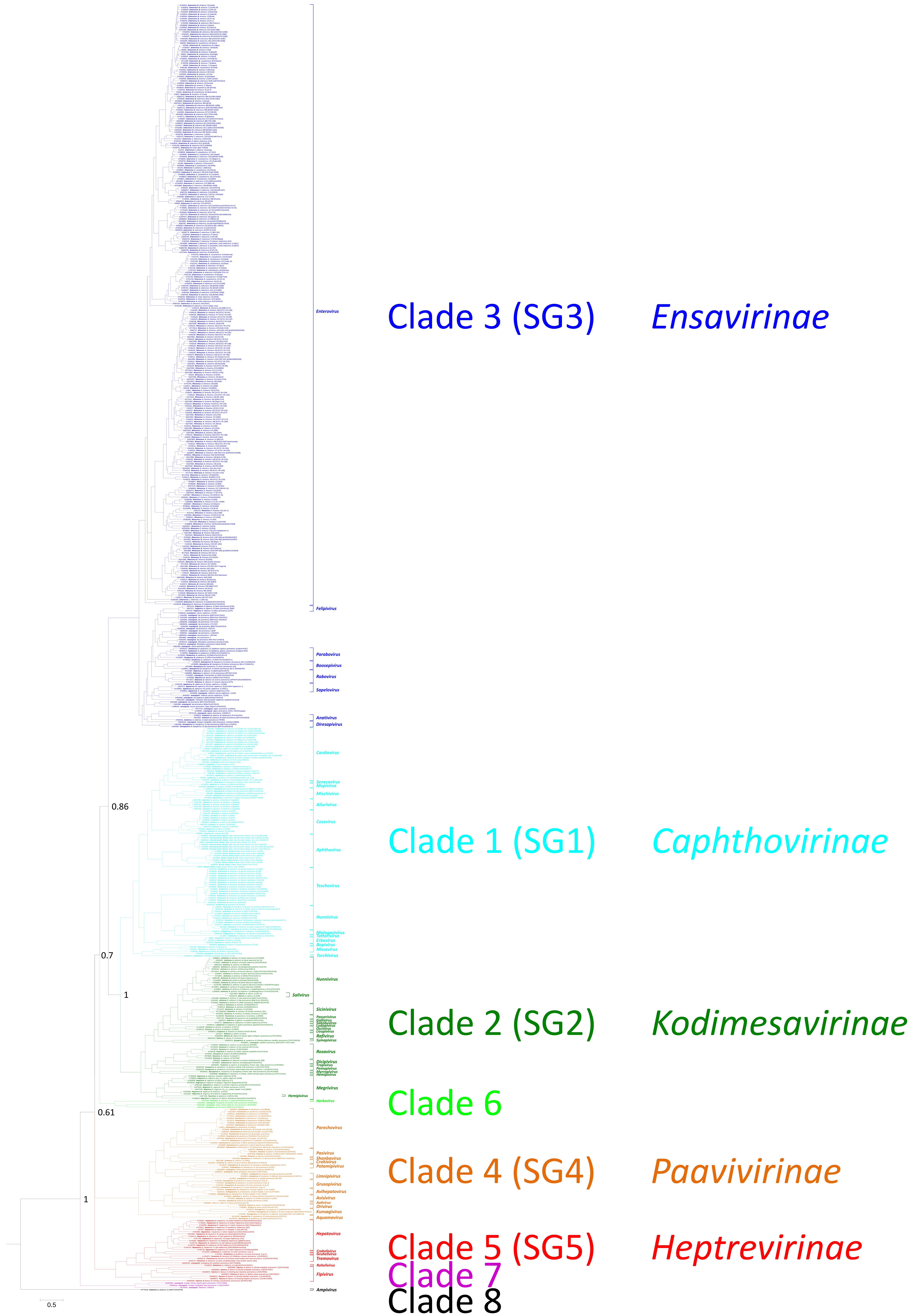
Clade 3 (SG3)
Ensavirinae
Clade 1 (SG1)
Caphthovirinae
Clade 2 (SG2)
Kodimesavirinae
Clade 6
Clade 4 (SG4)
Paavivirinae
Clade 5 (SG5)
Heptrevirinae
Clade 7
Clade 8
Enterovirus
Felipivirus
Parabovirus
Boosepivirus
Rabovirus
Sapelovirus
Anativirus
Diresapivirus
Cardiovirus
Senecavirus
Mupivirus
Mischivirus
Ailurivirus
Cosavirus
Aphthovirus
Teschovirus
Hunnivirus
Erbovirus
Bopivirus
Mosavirus
Torchivirus
Hunnivirus
Salivirus
Sicinivirus
Rosavirus
Megrivirus
Hemipivirus
Harkavirus
Porechovirus
Pasivirus
Limnipivirus
Grusopivirus
Avihepatovirus
Aquamavirus
Hepatovirus
Tremovirus
Fipivirus
Ampivirus
0.86
0.7
1
0.61
1
0.5
