# Supplementary figures and images for: A proposed division of the family Picornaviridae into subfamilies based on phylogenetic relationships and functional genomic organization
Source: Arch Virol. 2021 Aug 4;166(10):2927–35. doi: 10.1007/s00705-021-05178-9 (PMC8421316; doi:10.1007/s00705-021-05178-9)

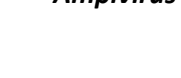

## Clade 1 (SG1) *Caphthovirinae*

Supplement: Supplementary file 2 — Supplementary file2Supplementary Fig. S2 Phylogenetic analysis of picornavirus P1 proteins. A total of 651 sequences representing the P1 region encoding the capsid proteins (5215 nt) of members of all known picornavirus species and types were analysed with MrBayes v3.2 (nucleotide substitution model GTR+G+I). Convergence was reached after 17 million generations. Sequences of clades 1 and 3 to 8 cluster in monophyletic clades (indicated in different colors). Presented are GenBank accession number, species name (in bold and italics), virus name/type, common name (if available, in round brackets), and strain designation (in square brackets). Posterior probabilities of major clades are presented. The scale indicates substitutions per nucleotide (PDF 787 KB) [file 705_2021_5178_MOESM2_ESM.pdf]

6 mio gen.  
GTR+G+I

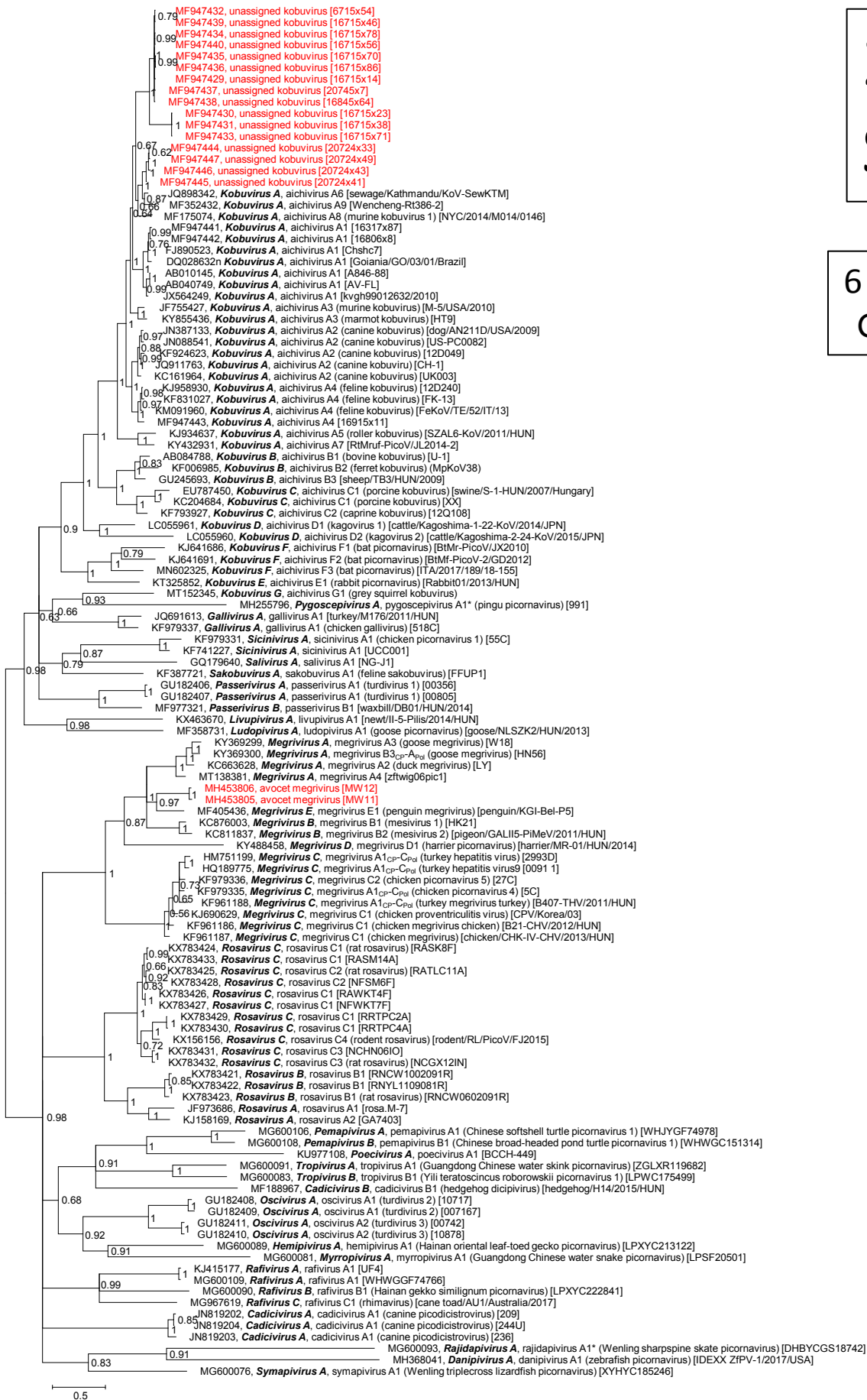

Supplement: Supplementary file 3 — Supplementary file3Supplementary Fig. S3 Phylogenetic analysis of supergroup 2 (Kodimesavirinae) 2B sequences. The alignment comprises 120 sequences of 22 genera, 38 species, 70 types, and 18 unassigned viruses. Tree inference was conducted with MrBayes, using the nucleotide substitution model GTR+G+I. Convergence was reached after 6 million generations. Unassigned viruses are shown in red. Presented are GenBank accession number, species name (in bold and italics), virus name/type, common name (if available, in round brackets), and strain designation (in square brackets) at the tips as well as posterior probabilities at the nodes. The scale indicates substitutions per nucleotide (PDF 295 KB) [file 705_2021_5178_MOESM3_ESM.pdf]

2A,  
SG3

7 mio gen.  
GTR+G+I

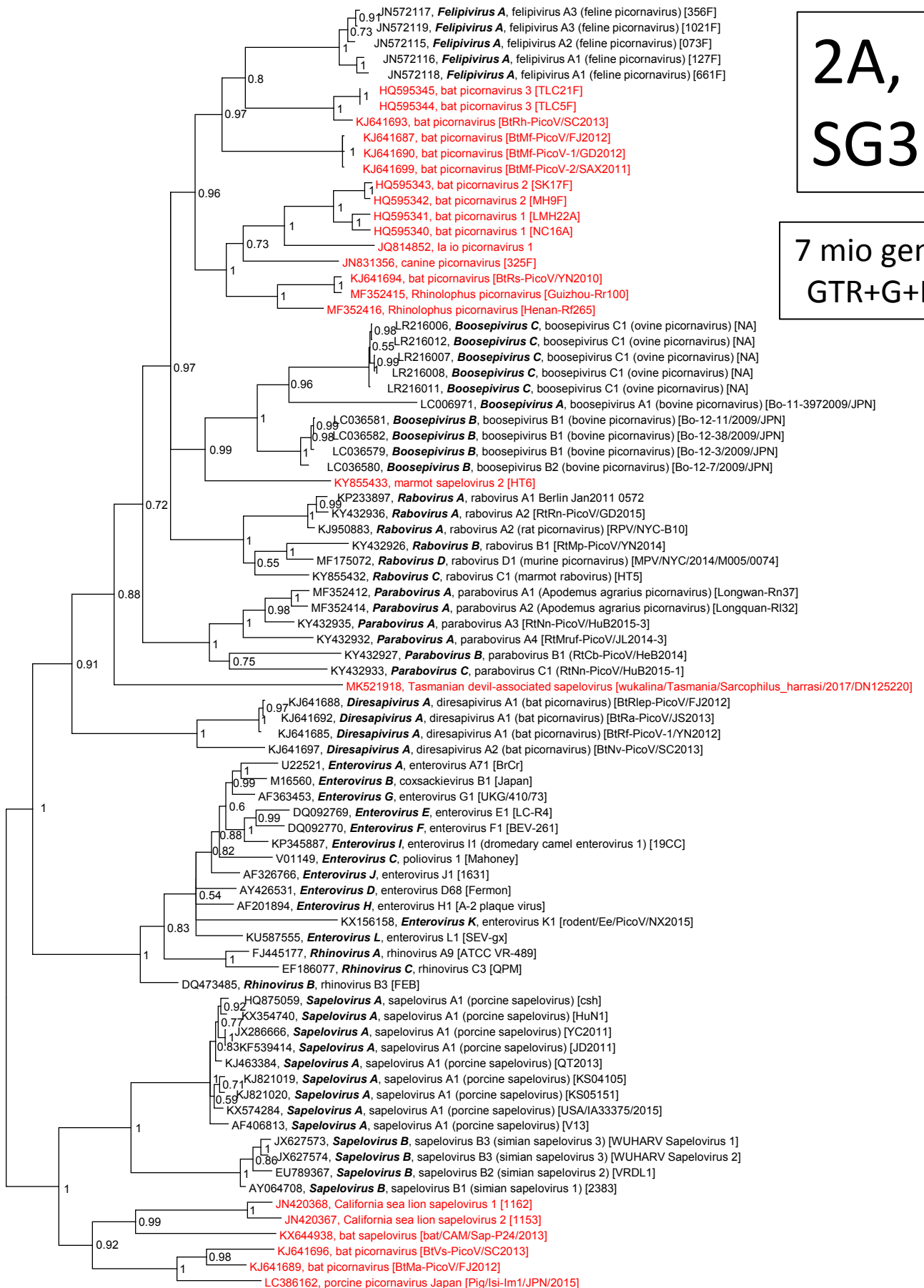

0.5

Supplement: Supplementary file 4 — Supplementary file4Supplementary Fig. S4 Phylogenetic analysis of supergroup 3 (Ensavirinae) 2A sequences. The alignment comprises 82 sequences of 8 genera, 30 species. Tree inference was conducted with MrBayes, using the nucleotide substitution model GTR+G+I. Convergence was reached after 7 million generations. Unassigned viruses are shown in red. Presented are GenBank accession number, species name (in bold and italics), virus name/type, common name (if available, in round brackets) and strain designation (in square brackets), at the tips as well as posterior probabilities at the nodes. The scale indicates substitutions per nucleotide (PDF 263 KB) [file 705_2021_5178_MOESM4_ESM.pdf]

2B,  
SG3

12 mio gen.  
GTR+G+I

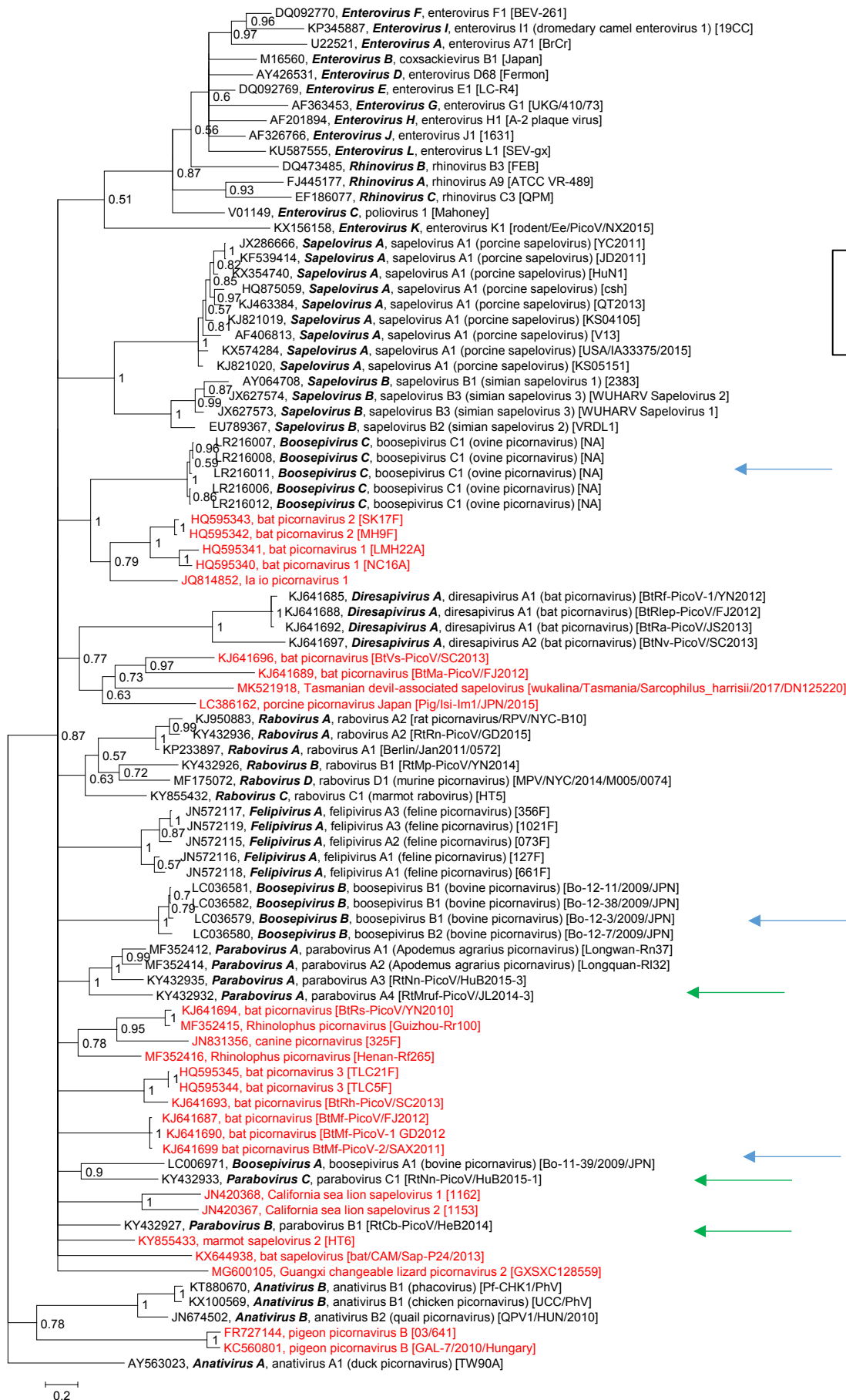

Supplement: Supplementary file 5 — Supplementary file5Supplementary Fig. S5 Phylogenetic analysis of supergroup 3 (Ensavirinae) 2B sequences. The alignment comprises 89 sequences of 8 genera, 30 species. Tree inference was conducted with MrBayes, using the nucleotide substitution model GTR+G+I. Convergence was reached after 12 million generations. Unassigned viruses are shown in red. Presented are GenBank accession number, species name (in bold and italics), virus name/type, common name (if available, in round brackets) and strain designation (in square brackets), at the tips as well as posterior probabilities at the nodes. The scale indicates substitutions per nucleotide. Blue and green arrows indicate inconsistent clustering of boosepiviruses and paraboviruses, respectively (PDF 259 KB) [file 705_2021_5178_MOESM5_ESM.pdf]

3A,  
SG3

7 mio gen.  
GTR+G+I

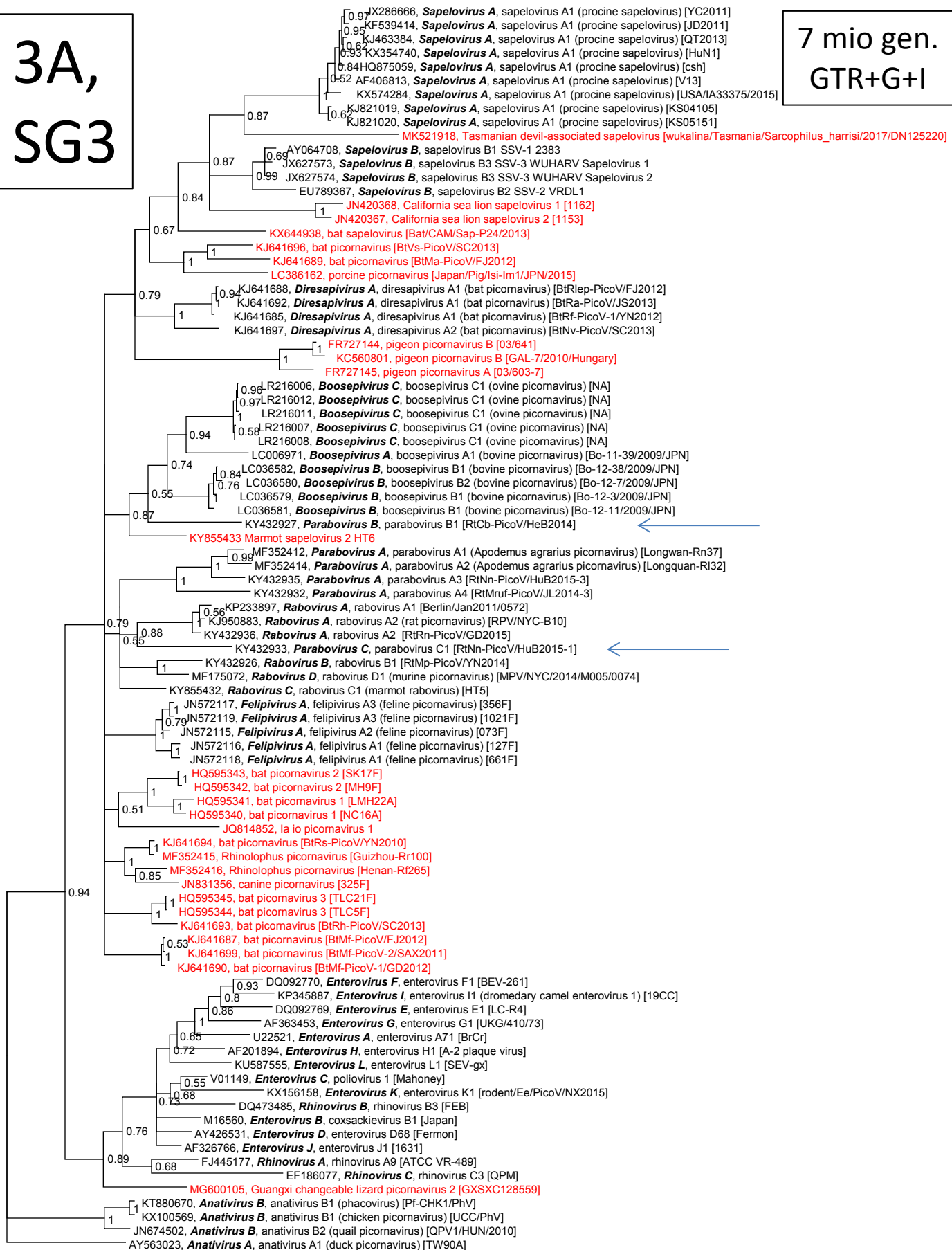

Supplement: Supplementary file 6 — Supplementary file6Supplementary Fig. S6 Phylogenetic analysis of supergroup 3 (Ensavirinae) 3A sequences. The alignment comprises 90 sequences of 8 genera, 30 species. Tree inference was conducted with MrBayes, using the nucleotide substitution model GTR+G+I. Convergence was reached after 7 million generations. Unassigned viruses are shown in red. Presented are GenBank accession number, species name (in bold and italics), virus name/type, common name (if available, in round brackets), and strain designation (in square brackets) at the tips as well as posterior probabilities at the nodes. The scale indicates substitutions per nucleotide. Blue arrows indicate inconsistent clustering of parabo B and C viruses (PDF 266 KB) [file 705_2021_5178_MOESM6_ESM.pdf]

2B,  
SG4

4 mio gen.  
GTR+G+I

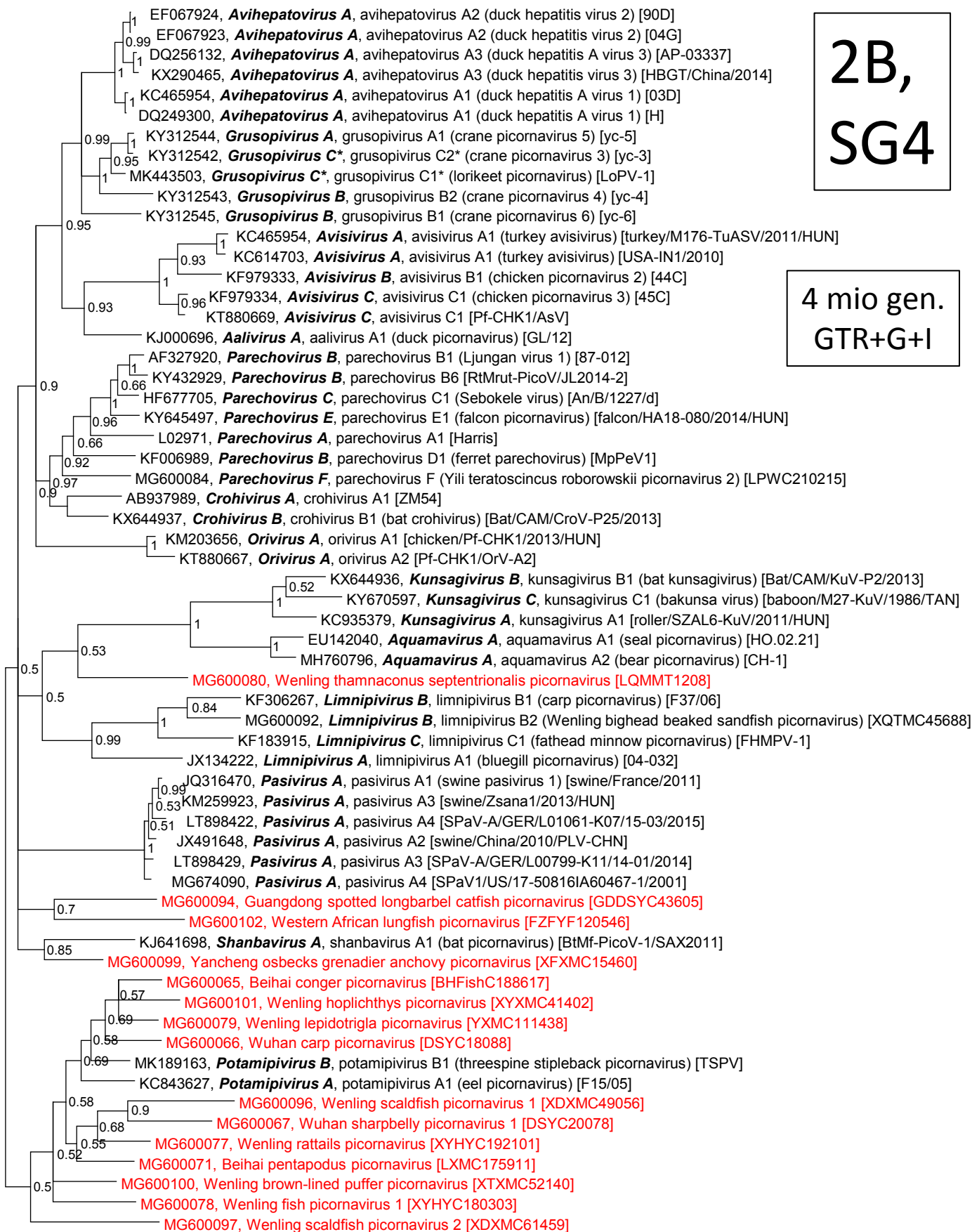

0.2

Supplement: Supplementary file 7 — Supplementary file7Supplementary Fig. S7 Phylogenetic analysis of supergroup 4 (Paavivirinae) 2B sequences. The alignment comprises 61 sequences of 13 accepted and proposed genera, 29 species. Tree inference was conducted with MrBayes, using the nucleotide substitution model GTR+G+I. Convergence was reached after 4 million generations. Unassigned viruses are shown in red. Presented are GenBank accession number, species name (in bold and italics), virus name/type, common name (if available, in round brackets), and strain designation (in square brackets) at the tips as well as posterior probabilities at the nodes. The scale indicates substitutions per nucleotide (PDF 245 KB) [file 705_2021_5178_MOESM7_ESM.pdf]

**P1**

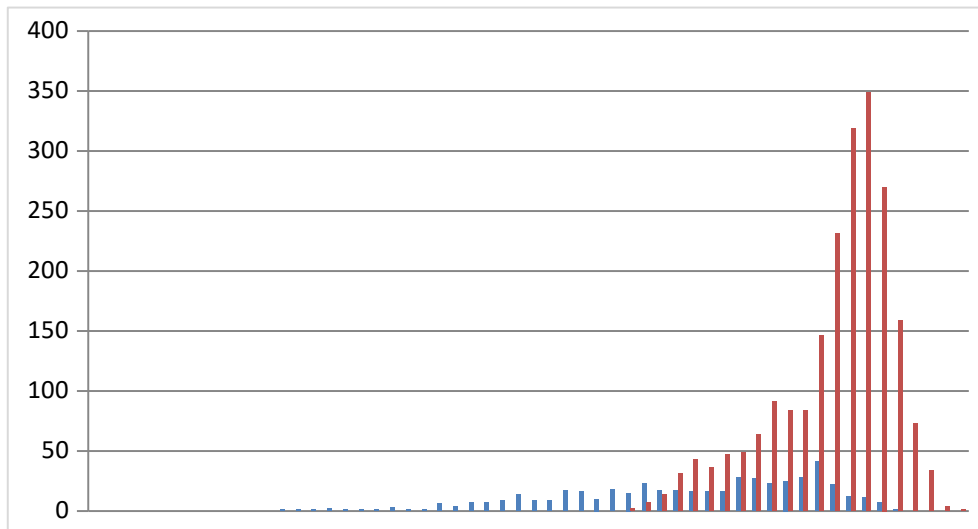

**3CD**

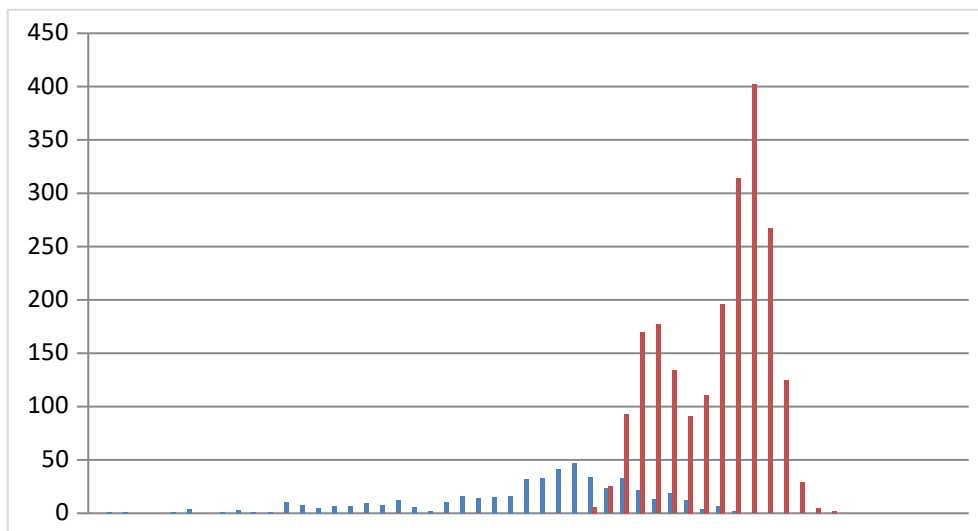

**3D**

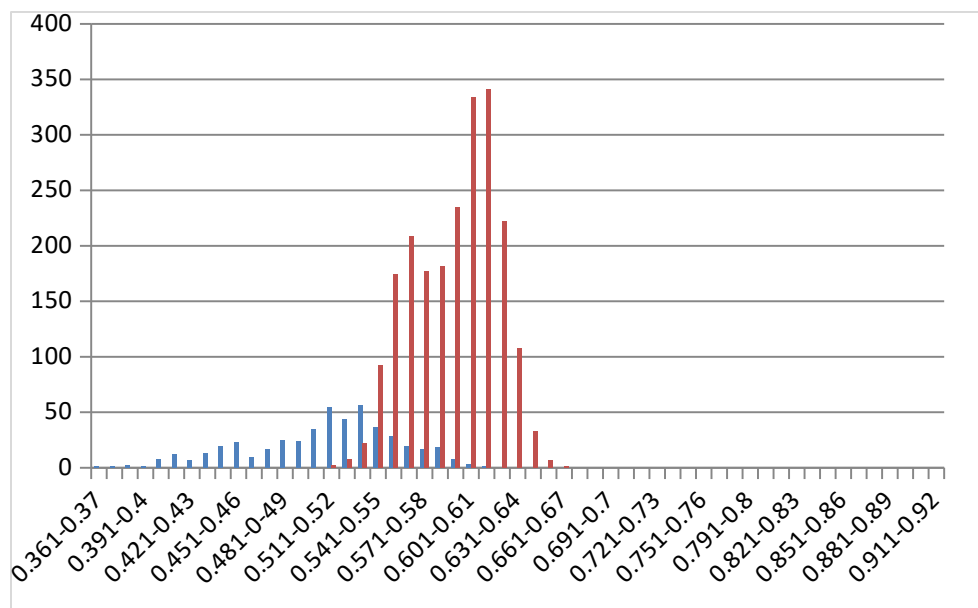

within-subfamily comparisons

between-subfamily comparisons

Supplement: Supplementary file 8 — Supplementary file8Supplementary Fig. S8 Frequency distribution of pairwise amino identity scores. P1, 3CD, and 3D proteins were compared. A total of 155 picornavirus P1 sequences and 156 3CD and 3D sequences were grouped into 68 genera. The data sets were completed with six sequences of unassigned viruses and were used for the estimation of pairwise amino acid identity scores (PDF 37 KB) [file 705_2021_5178_MOESM8_ESM.pdf]
